# “But Will It Last?”: Examining How Pharmacy Staff Perceptions Influence Beliefs About the Sustainability of a Pharmacy-Based Intervention Targeting Older Adult Over-the-Counter (OTC) Medication Misuse

**DOI:** 10.3390/pharmacy13060174

**Published:** 2025-12-01

**Authors:** Aaron M. Gilson, Katherine G. Moore, Stephanie M. Resendiz, Emily L. Hoffins, Shiying Mai, Jamie A. Stone, Michelle A. Chui

**Affiliations:** 1 Sonderegger Research Center for Improved Medication Outcomes, University of Wisconsin-Madison School of Pharmacy, Madison, WI 53705, USA; aaron.gilson@wisc.edu; 2 Chui SAMS (Systems Approach to Medication Safety) Lab, University of Wisconsin-Madison School of Nursing, Madison, WI 53705, USA; katherine.moore@wisc.edu (K.G.M.); jamie.stone@wisc.edu (J.A.S.); 3 Division of Social and Administrative Sciences, University of Wisconsin-Madison School of Pharmacy, Madison, WI 53705, USA; smresendiz@wisc.edu (S.M.R.); smai5@wisc.edu (S.M.); 4 Department of Pharmacy Systems, Outcomes and Policy, University of Illinois Chicago, Chicago, IL 60612, USA; ehoffins@uic.edu

**Keywords:** sustainability, senior safe, OTC misuse, older adults, pharmacy staff, pharmacists, pharmacy technicians, confidence, survey

## Abstract

Sustaining a well-designed healthcare intervention justifies the resources allocated during its conceptualization and implementation and maximizes its clinical benefits, but staff influences on sustainment have been studied insufficiently. This study evaluates the effects of pharmacy staff (i.e., pharmacists/technicians) perceptions about the sustainability of Senior Safe^TM^, a U.S. pharmacy-based intervention to reduce older adult over-the-counter (OTC) medication misuse. Three months after introducing Senior Safe into 67 pharmacies in a large Midwestern health-system, all pharmacy staff (N = 279) received a survey invitation. Fifty-nine pharmacists and 94 technicians completed the survey. Using logistic regression modeling for the 14 belief-based survey items, and staff roles (pharmacist or technician), the final factors significantly predicting staff views that Senior Safe was sustainable were as follows: perceiving Senior Safe as well-integrated into leadership operations (OR = 5.606, *p* < 0.001) and believing the intervention reduced OTC misuse (OR = 8.217, *p* < 0.001). Also, technicians were more confident than pharmacists about Senior Safe’s sustainment and its OTC misuse reduction success. Overall, an intervention’s sustainability relies on those using it. Since the principal predictor of maintaining Senior Safe was its perceived effectiveness, increasing staff buy-in and awareness of an intervention’s benefits may be central to its long-term viability. With an aging U.S. population, sustainable solutions to older adult medication misuse remain critical.

## 1. Introduction

Modern-day workflow in community pharmacy is characterized by a high workload that can result in staff burnout [1,2,3]. Given the typical number of stressors in everyday pharmacy practice, additional workload responsibilities can result in increased fatigue and frustration [4], which can exacerbate the frequency of medication-related errors [5]. Such effects can have a detrimental impact on patient safety [6].

Historically, healthcare interventions commonly require additional professional responsibilities [7], which can stress a system’s traditional workflow. This situation can also apply to research- and evidence-based interventions that are imposed on a system to improve patient safety [8]. Even when such interventions are implemented and demonstrate signals of short-term effectiveness, there has typically been little consideration for maintaining the intervention beyond the scope of its initial evaluation [9]. Even with the increased focus on research evaluations about intervention effectiveness targeting patient safety, evidence from systematic reviews suggests that study design characteristics [10] often make it difficult to determine utility.

Another factor influencing successful intervention adoption and maintenance is acceptability by, among others, the healthcare professionals who implement the intervention [11]. For example, when a pharmacy introduces a new intervention, but pharmacy staff do not have complete buy-in, intervention viability can be sacrificed. However, pharmacy staff can facilitate intervention support, such as through their perceptions of system-level support for the intervention, which can be affected by having a sense of leadership/managerial backing [12,13,14,15,16] (see Section 1.1 for examples of organizational backing related to the intervention specific to this paper).

Research also suggests that staff members’ commitment to an intervention is influenced by the perception that it “would have any real effect on behavioral change” (p. 10) [17]. That is, organizational stakeholders’ confidence that an intervention will likely achieve its patient safety goals can increase their commitment to that intervention.

Despite evidence that these factors are important, pharmacy staff are rarely asked for their input or about supporting intervention design or adoption [18]. Even when data collection has involved pharmacy staff, feedback was generally collected from pharmacists but tended to exclude technicians [19,20,21]. Given the critical role that technicians also play in supporting pharmacy operations and patient care [19], failure to gather their perspectives misses an opportunity to more completely inform pharmacy-based interventions.

Pharmacy staff perspectives about an intervention can be systematically collected to inform its development, prior to its adoption in the pharmacy, and even after its adoption and use for a period of time [15,22,23,24]. Ideally, early in the intervention conceptualization process, pharmacy staff can offer guidance about how to best design an intervention to achieve its long-term goals [15], and do so without exacerbating workload burden, which ultimately can enhance confidence that the intervention is both effective and sustainable.

### 1.1. A Pharmacy-Based Intervention: Senior Safe^TM^

A pharmacy staff-informed approach was used when designing Senior Safe^TM^, a U.S. evidence-based intervention created to reduce older adult (age 65+) misuse of certain over-the-counter (OTC) products [25] without generally exacerbating workload [15]. The OTC products that were relevant to Senior Safe involved medications used to treat cough/cold/allergy, pain, or sleep symptomology. Senior Safe was implemented into 67 pharmacy sites comprising a large Midwestern healthcare system and represented a large-scale health system-level intervention. Senior Safe attempted to prevent older adults from misusing OTC medications by redesigning the pharmacy’s OTC aisles [25,26]. Specially designed signage was placed on the OTC shelves to distinguish low- and high-risk OTC products, distinguished by Beer’s criteria [27], that were relevant to this intervention [26]. Distinct signage was located on the shelves alongside designated “safer” products (represented by a green banner) or “high-risk” products (represented by a red stop sign). Alternatively, OTCs with the highest risk were moved behind-the-counter (BTC) and replaced by BTC signage. Senior Safe succeeded in immediately reducing older adult OTC misuse, using a randomized control trial design [26], as well as three months afterward [28], without increasing burden on staff workload [15].

Throughout Senior Safe conceptualization and creation, pharmacists, technicians, and older adults (the population for which Senior Safe was developed) were able to provide feedback through a series of semi-structured focus groups [29]. In fact, it was the focus groups of pharmacists and technicians that led to the consideration of BTC signage—these stakeholders strongly believed that it was an important tool for enhancing older adult safety around particularly high-risk OTC medications. Systematically gathering input from intervention stakeholders is an important characteristic for developing healthcare interventions [7]. Ongoing efforts were made throughout intervention development to ensure organizational leadership integration (e.g., conceptual buy-in, integration into system mission, pharmacy staff support, and participation in activities to maximize intervention utility). In addition, a critical component of the Senior Safe adoption process was the use of systematic educational training—including the use of a 15 min long standardized video— with pharmacy staff to inform them of the goals of, and their responsibilities related to, this intervention, including the need to engage with older adult patients around medication safety issues [29]. The purpose of both the ongoing pharmacy staff feedback solicitation and the training was to generate an understanding of and commitment to Senior Safe and its patient safety goal. This approach was believed to strengthen pharmacy staff’s desire to maintain the intervention after project completion.

### 1.2. Objectives

To understand the beliefs of pharmacy staff that support confidence in Senior Safe’s long-term viability, a survey was sent to pharmacists and technicians who worked with adopting and enacting Senior Safe in their pharmacies. The survey was designed to evaluate pharmacy staff perceptions about various aspects of leadership support, encountering and addressing issues related to intervention adoption, and their preparation for their role in enacting Senior Safe, as well as Senior Safe’s effectiveness at reducing OTC medication misuse and the confidence in its activity in five years. Statistical analyses were used to determine the predictive importance of these factors on pharmacy staff assurance that Senior Safe is maintainable.

## 2. Materials and Methods

### 2.1. Sample Recruitment

Three months following the final implementation of Senior Safe in a large Midwestern healthcare system, its regional pharmacy manager sent invitations via email for survey participation (in April 2024). All 279 staff members (82 pharmacists and 197 technicians)—working at either traditional pharmacy or remote dispensing (RD) sites within the health system—received the survey. As opposed to traditional pharmacy sites, RD sites are staffed by at least one technician, and medication consultations are performed via TelePharm with a pharmacist from a nearby pharmacy. The survey, measuring staff perspectives about various aspects of Senior Safe implementation, was adapted from existing surveys [30,31,32] and constructed in REDCap [33,34].

The regional pharmacy manager sent an initial email containing the invitation (16 April 2024) and two reminder emails (18 April and 22 April 2024). The survey was closed one month after the second reminder email was sent. Pharmacy staff received a $10 Amazon gift card for participation.

### 2.2. Sustainability Survey

Research team members categorized the 14 belief-based content items in this survey (Supplementary Materials File S1) into four separate thematic domains, with one item (including two branching sub-items) being omitted because it was a knowledge question and did not represent a respondent’s confidence level. The resulting domains were:

(1) Pharmacy Leadership Support (5 items, questions 1–5): beliefs about the ways in which Pharmacy Leadership (including Manager of Pharmacy Retail Operations or the Director of Retail Pharmacy Operations) have supported Senior Safe,
(2) Preparedness for Role in Senior Safe (7 items, questions 6, 8–13): beliefs about whether local site leader supports staff in maintaining Senior Safe, and pharmacy staff understanding of Senior Safe and their role in its adoption,
(3) Effectiveness of Senior Safe (1 item, question 15): beliefs about Senior Safe’s ability to reduce OTC misuse, and
(4) Long-Term Effectiveness (the single dependent variable, question 14): beliefs that Senior Safe would still be active in five years.

Although the Sustainability survey contained a number of demographic and pharmacy site items, the only pharmacy staff demographic characteristic that was evaluated was pharmacy role (i.e., whether the respondent was a pharmacist or technician). This approach was decided based on the authors’ interest in illustrating the voices of pharmacists and pharmacy technicians separately, as well as to limit the number of items to include in the model in an attempt to preserve analytic power.

All belief-based survey content items were initially scored on a four-point Likert scale relating to the respondent degree of confidence (i.e., “not at all,” “somewhat,” “very much,” and “unsure”). The survey items were subjected to cognitive interviewing by healthcare system collaborators and two pharmacy staff to ensure readability and that the language and professional titles used in the items were interpretable by all respondents.

### 2.3. Analysis

Given the survey items’ original four-point Likert scaling, and for the purpose of these analyses, survey responses were dichotomized into “More Confident” (comprising “very much” responses) and “Less Confident” (i.e., “somewhat,” “not at all,” and “unsure”). Responses indicated as “Prefer not to answer” were coded as missing data. As a result, all survey variables maintained for analyses were coded as binomial.

A three-phase logistic regression modeling approach was used to determine the influence of survey items on the dependent variable (staff’s confidence that Senior Safe would still be active in five years). Phase 1 represented a series of logistic regression models to identify the significant effects of the number of variables representing each thematic domain. Phase 2 created a model containing all significant variables resulting from the Phase 1 analyses, which was expected to reflect at least some aspects of all domains, representing a multi-domain model. Finally, Phase 3 compared the Akaike’s Information Criterion (AIC) and Bayesian Information Criterion (BIC) values derived for the multi-domain model to the AIC and BIC values for the individual domain models. Such a comparison demonstrated whether the multi-domain model attained a greater goodness of fit and had a higher predictive strength than the other variable domains, while accounting for the different number of predictors in each model. A formal power analysis was not appropriate due to the exploratory nature of this analysis. IBM SPSS Statistics Version 31.0.0.0 [35] was used for all analyses, employing two-tailed *p* < 0.01 significance values.

## 3. Results

### 3.1. Descriptive Analyses

Of the 279 staff who were sent the survey invitation, 168 responded. Of these responses, eight were excluded for either responding to fewer than 50% of items or providing no information about their setting or role and no usable demographic information, while another respondent was removed for answering “unsure” for all survey questions and providing inadequate demographic data. The final sample consisted of 160 respondents, resulting in a 57.4% response rate.

### 3.2. Pharmacy Staff Roles

The sample consisted of more technicians (n = 94) than pharmacists (n = 59), but seven respondents did not report their pharmacy role. The sample pharmacist-to-technician ratio was higher, and therefore more equal, than the ratio characterizing the entire pharmacy staff population within the healthcare system (0.628 for the sample compared to 0.416 for the population), which is typical for pharmacy practice [36].

### 3.3. Survey Item Descriptives

Table 1 shows that, for all pharmacy staff, a two-thirds majority expressed greater confidence in the Pharmacy Staff Leadership domain about Senior Safe being part of the health system’s mission and having leadership support its goals. This same majority level was also found in the Role Preparedness domain for the variables of having more confidence in understanding intervention goals and being committed to achieving those goals. In addition, in the Pharmacy Staff Leadership domain, about 54% of all respondents were confident that Senior Safe was well-integrated into leadership operations. Fewer than 50% of pharmacy staff reported more confidence for all other survey items. However, no confidence rate showed more than about a 5% deviance from 50%, except for pharmacy staff leadership asking staff about what does/does not work, with only 24.4% having reported more confidence.

When considering differences according to pharmacy role, technicians expressed greater confidence than pharmacists on many more survey items (11 items compared to three items, respectively) (Table 1). Notably, more technicians showed greater confidence than pharmacists relating to Senior Safe being well-integrated into leadership operations (+12.1%), leadership supporting Senior Safe goals (+13.7%) and clearly communicating those goals (+20.1%), leadership asking pharmacy staff about what does/does not work (+19.4%), that the pharmacy supervisor supports staff in maintaining Senior Safe (+10.1%), and that Senior Safe reduces OTC misuse (+15.5%) and would last over the next five years (+16.5%). These items represent the technicians’ largest divergence from pharmacists.

Alternatively, pharmacists were more confident than technicians only about their understanding of Senior Safe goals (+5.0), feeling adequately trained for Senior Safe (+5.7%), and feeling empowered to suggest what does and does not work with Senior Safe (+17.8%).

### 3.4. Statistical Analyses

Logistic regression models for the variables in each domain were computed initially. Regression results identified respondents’ confidence levels about Senior Safe lasting. For each comparison, each categorical variable’s reference category was “less confident,” generating predictive effects related to greater confidence.

### 3.5. Single Thematic Domain Modeling

Table 2 provides the results for each of the three thematic domains of independent variables, as well as the pharmacy role demographic characteristic, which were modeled independently to identify significant factors that were then included in a comprehensive multivariate model (see Section 3.6). Of the five variables comprising the Pharmacy Staff Leadership domain, believing that Senior Safe was well-integrated into leadership operations predicted confidence about the intervention being active in the next five years (OR = 4.961, *p* < 0.001). Although the Role Preparedness domain contained more variables than the Leadership domain, only one significant variable was again identified: Being committed to achieving intervention goals (OR = 10.798, *p* = 0.004). The Senior Safe Effectiveness domain, comprised solely of the belief that Senior Safe helps reduce OTC medication misuse, achieved significance (OR = 12.893, *p* < 0.001). Finally, the model representing pharmacy staff role was not significant at the *p* < 0.01 level. Of these individual domain models, the smallest AIC and BIC values were 12.869 and 18.982, respectively, for the single-variable Senior Safe Effectiveness domain.

### 3.6. Multi-Domain Multivariate Modeling

A multi-domain multivariate model, comprising the three significant variables from the individual domain models, was constructed to determine their resulting significance when controlling for the other previously significant variables (Table 3). Within this model, two variables maintained their significance related to Senior Safe sustainability: (1) how well the intervention was integrated into the operations of leadership (OR = 5.606, *p* < 0.001), and (2) whether staff believed that the intervention successfully reduced OTC misuse (OR = 8.217, *p* < 0.001). As with the domain-level analysis, believing that Senior Safe effectively reduces older adult OTC misuse was the strongest predictor in the confidence of Senior Safe being maintained in the pharmacy. This multi-domain model had AIC and BIC values of 30.363 and 42.537, respectively, which were the smallest values of any computed multivariate model. This result suggests an improvement in the fit of the multi-domain model over all more complex single-domain models, but not those models representing only one variable.

## 4. Discussion

Senior Safe was a pharmacy-based intervention designed with pharmacy staff acceptability in mind, actively involving them during its conceptualization, development, and adoption [15]. Given pharmacy staff contributions to Senior Safe development, it was important to garner feedback from both pharmacists and technicians about their experiences with the intervention, as well as to investigate whether their beliefs about various aspects of Senior Safe, such as leadership support or intervention effectiveness, were associated with confidence in its long-term viability. Study results suggest that pharmacy staff’s confidence in Senior Safe lasting was dependent on (1) thinking that it was well-integrated into leadership’s operations and (2) believing that it successfully reduced OTC misuse. Therefore, it is possible that, when implementing a novel pharmacy program, identifying staff’s confidence in these content areas can help pinpoint which staff may need extra support and which staff members might help champion ongoing adoption efforts [14,37].

Notably, confidence in Senior Safe’s effectiveness at reducing OTC misuse for older adults had the greatest predictive value of all analyzed variables in the individual models. In the multi-domain model, pharmacy staff who more strongly believed in the intervention’s ability to achieve its primary objective were eight times more likely to envision Senior Safe being active in five years, even when accounting for other factors in the regression model. This finding might be accounted for, at least in part, by prior research showing that the Senior Safe intervention effectively reduced older adult misuse of higher-risk OTC products [26]. Communicating these effectiveness findings was foundational to the pharmacy staff training in preparation for broad Senior Safe adoption. The model finding also reinforces prior investigation into the influence of perceptions about the effectiveness of system change [38], where informing staff about the potential impact of a system change can facilitate its adoption or successful maintenance [39,40,41]. Whenever possible, it seems reasonable to inform pharmacy staff about an intervention’s prospective benefit to patients, as a means to promote buy-in and confidence in its long-term viability. Cumulatively, Senior Safe benefits from having both immediate and short-term effectiveness at reducing older adults’ OTC medication misuse, as well as solid pharmacy staff perceptions about its effectiveness at achieving its primary goal.

When pharmacy staff felt that Senior Safe was well-integrated into pharmacy leadership operations, as a sign of organizational commitment to the intervention, they had more confidence that it would be a lasting service. This finding also could reflect the tendency for staff’s beliefs around overall organizational commitment to play a role in mitigating burnout, which can promote intervention longevity due to decreased staff work burdens [3]. This interpretation conforms to prior studies supporting organizational commitment as important for motivating staff to strive to achieve the goals of the healthcare organization in which they work [42].

It is interesting that the two statistically significant items held more explanatory power about Senior Safe sustainability than other seemingly important system-related factors [43,44], such as the intervention representing the organization’s mission, having leadership support the intervention goals and offering clear communications about it, commitment to intervention goals, and being adequately trained to fulfill the intervention objectives. Although bivariate analyses initially revealed significant effects at the *p* < 0.01 level for each of these variables (post hoc analyses, not reported), their predictive effects were mitigated when controlled for the influences of other domain factors or, when in the final model, controlling for leadership-intervention integration and the intervention’s potential to achieve its goal. This ultimate parsimony supports the need to examine the influence of system factors within a multivariate statistical framework, to determine a factor’s effect after accounting for the simultaneous influences of other system characteristics.

In the single-domain model, no statistically significant difference emerged between pharmacists and technicians, at least at the *p* < 0.01 level. As a result, this regression modeling finding seems to suggest that technicians evidenced a generally similar degree of confidence, when compared to pharmacists, that Senior Safe would remain active in their pharmacy in five years. However, examining proportional differences in confidence levels between pharmacy staff roles revealed a notable discrepancy regarding Senior Safe sustainability, with 16.5% more technicians than pharmacists having more confidence in intervention sustainability. A similar degree of confidence difference was reported about Senior Safe effectiveness, as well as for a number of other survey items. Cumulatively, these findings reinforce the importance of considering the potential for monitoring pharmacist/technician differences when examining pharmacy staff perceptions about intervention viability. When technicians are anticipated to have specific intervention-related responsibilities, as with Senior Safe, it is valuable to capture their feedback throughout the entire intervention development and adoption process.

It is also important to note that, for half of all survey items, fewer than 50% of all pharmacy staff respondents reported more confidence. In order of decreasing proportion of confidence, those items were: Pharmacy staff leadership clearly communicates intervention goals (49.4%), being adequately trained for the intervention (48.7%), feeling empowered to suggest what does/does not work (46.9%), having clearly defined intervention responsibilities (46.3%), believing that Senior Safe helps reduce OTC medication misuse (45.6%), believing that Senior Safe will be active in five years (44.4%), and having pharmacy staff leadership asking staff about what does/does not work (24.4%). Although all but one of these proportions could not be described as representing a sizeable minority, it is noteworthy that some of these issues were foundational to the pharmacy staff training prior to Senior Safe implementation, and would be expected to have influenced their confidence about these topics. Such issues include the communication of Senior Safe’s goals and the prospect of its long-term enactment, the delineation of staff responsibilities, and adequate training, even though a training program was required for participation in the intervention. The implications of these findings are exacerbated by the fact that there were clear distinctions between pharmacists’ and technicians’ confidence levels, with pharmacists reporting less confidence for these and most other items. It, therefore, seems incumbent on project researchers to investigate the reasons why topics covered in pharmacy staff training may not translate into greater confidence after the intervention has been in effect, or to elicit feedback from survey respondents to substantiate their lack of confidence about certain items. Researchers then could focus on potential communication or messaging improvements about the purpose of Senior Safe and the various ways in which pharmacy staff can maximize its effectiveness. The researchers may also investigate pharmacy staff’s initial beliefs about Senior Safe characteristics, before the intervention is implemented, to counter potential misperceptions and enhance their receptivity as it is adopted.

## 5. Limitations

Despite these analyses yielding interesting findings about factors that can affect system-change, a number of limitations characterize this study. First, this study involved pharmacy sites from a single large healthcare system, so results may not be generalizable to other pharmacy settings or to other health organizations. Future research introducing Senior Safe into other pharmacy settings, while using the same survey tool to assess sustainability perceptions, would help enhance the generalizability of the findings. Second, study data were derived from a self-report survey, which introduces potential bias. Third, nonresponse bias is a possibility, as non-respondents might have had differing degrees of confidence compared to those who chose to respond. However, the resulting sample represented 57.4% of the pharmacy staff population, which is higher than the average paid online survey response rate [45], increasing the likelihood of better representativeness. Fourth, the sustainability survey was adapted from existing tools [30,31,32] but not validated for the purpose of this study. Fifth, the survey was distributed to the staff from all healthcare system pharmacy sites, which included three sites (4.5% of all sites) that did not implement Senior Safe due to being under construction or out-of-state. It is possible that a few surveys were completed by staff from pharmacies that did not adopt the intervention. However, only one completed survey was excluded from analyses because all of the responses were “unsure,” which was interpreted by researchers as suggesting that the respondent worked in a pharmacy having no involvement with Senior Safe. Sixth, this analysis represented a single point-in-time assessment approach. Future research could be designed to evaluate longer-term sustainability perceptions in the face of the healthcare system’s periodic communication of Senior Safe principles to its pharmacy staff. Finally, the power analysis calculated for this overall project was based on the primary study objective—Senior Safe’s effect on older adult OTC medication misuse—which was assessed using a randomized clinical trial design and did not relate to the analysis of a different dependent variable. Given this, the cumulative number of variables selected for this exploratory regression modeling process conformed to the general recommendation of 10 cases per independent variable [46], with confidence in the results further increased by using a *p* < 0.01 level to identify statistical significance. As a result, the two statistically significant variables from the final multi-domain model (e.g., organizational leadership integration and intervention effectiveness) can likely be considered valid predictors of perceptions about Senior Safe sustainment and have important implications.

## 6. Conclusions

Pharmacists and technicians who had experience with implementing and maintaining Senior Safe functioning, and generally perceived it to be well-integrated into pharmacy leadership operations and were convinced of its effectiveness, were more likely to most strongly believe in its long-term viability. Importantly, the factor with the greatest predictive strength was pharmacy staff’s conviction about Senior Safe’s effectiveness at reducing older adult OTC medication misuse. A clear implication of these findings is that confidence in the long-term maintenance of an intervention, such as Senior Safe, may be strengthened through conscientious efforts at organizational integration and convincing pharmacy staff of the intervention’s potential positive impact on patient care. Together, pharmacists and technicians can offer indispensable, yet potentially distinct, guidance for the conceptualization, refinement, finalization, and adoption of interventions. Pharmacy staff are essential to the continued successful use of pharmacy-based interventions, just as they have been with Senior Safe.

## Figures and Tables

**Table 1:** Pharmacy Staff Confidence Levels for Sustainability Survey Items.

	More Confidence (n, %)			Less Confidence (n, %)		
Items in Each Thematic Domain	Overall (n = 160)	Pharmacists (N = 59)	Technicians (N = 94)	Overall (n = 160)	Pharmacists (n = 59)	Technicians (N = 94)
**Pharmacy Staff Leadership**						
Senior Safe is part of the mission of the health system	106 (66.3)	37 (62.7)	64 (68.1)	54 (33.8)	22 (37.3)	30 (31.9)
Senior Safe is well-integrated into leadership operations	86 (53.8)	28 (47.5)	56 (59.6)	74 (46.3)	31 (52.5)	38 (40.4)
Leadership supports Senior Safe goals	118 (73.8)	39 (66.1)	75 (79.8)	42 (26.3)	20 (33.9)	19 (20.2)
Leadership clearly communicates Senior Safe goals	79 (49.4)	22 (37.3)	54 (57.4)	79 (49.4)	36 (61.0)	39 (41.5)
Leadership asks staff about what does/does not work about Senior Safe	39 (24.4)	8 (13.6)	31 (33.0)	117 (73.1)	49 (83.1)	62 (66.0)
**Preparedness for Role in Senior Safe**						
Supervisor supports staff in maintaining Senior Safe	113 (70.6)	38 (64.4)	70 (74.5)	45 (28.1)	19 (32.2)	24 (25.5)
There are usually enough staff to achieve Senior Safe goals	84 (52.5)	29 (49.2)	53 (56.4)	74 (46.8)	30 (50.8)	40 (42.6)
I understand Senior Safe goals	115 (71.9)	45 (76.3)	67 (71.3)	45 (28.1)	14 (23.7)	27 (28.7)
I am committed to achieving Senior Safe goals	125 (78.1)	45 (76.3)	76 (80.9)	33 (20.6)	14 (23.7)	16 (17.0)
I have clearly defined Senior Safe responsibilities	74 (46.3)	247 (45.8)	45 (47.9)	86 (53.8)	32 (54.2)	49 (52.1)
I am adequately trained for Senior Safe	76 (48.7)	31 (52.5)	44 (46.8)	80 (50.0)	27 (45.8)	48 (51.1)
I feel empowered to suggest what does/does not work with Senior Safe	75 (46.9)	35 (59.3)	39 (41.5)	79 (49.4)	24 (40.7)	51 (54.3)
**Senior Safe Effectiveness**						
Senior Safe helps reduce OTC medication misuse	73 (45.6)	21 (35.6)	48 (51.1)	87 (54.4)	38 (64.4)	46 (48.9)
**Senior Safe Sustainability (Dependent variable)**						
I am confident Senior Safe will still be active in five (5) years	71 (44.4)	21 (35.6)	49 (52.1)	86 (53.8)	38 (64.4)	43 (45.7)

**Table 2:** Multivariate Logistic Regression Effects for Individual Thematic Domain Models.

			99% CI	
	Odds Ratio	Significance Level	Lower	Upper
**Pharmacy Staff Leadership** **(AIC = 54.16, BIC = 72.11)**				
Senior Safe is part of the mission of the health system				
Less confident	--	--	--	--
More confident	1.433	0.498	0.365	5.625
Senior Safe is well-integrated into leadership operations				
Less confident	--	--	--	--
More confident	4.961	**<0.001**	1.479	16.644
Leadership supports Senior Safe goals				
Less confident	--	--	--	--
More confident	0.925	0.897	0.195	4.375
Leadership clearly communicates Senior Safe goals				
Less confident	--	--	--	--
More confident	1.376	0.505	0.400	4.732
Leadership asks staff about what does/does not work with Senior Safe				
Less confident	--	--	--	--
More confident	2.127	0.147	0.556	8.131
**Preparedness for Role in Senior Safe** **(AIC = 98.333, BIC = 122.202)**				
Supervisor supports staff in maintaining Senior Safe				
Less confident	--	--	--	--
More confident	2.807	0.046	0.742	10.629
There are usually enough staff to achieve Senior Safe goals				
Less confident	--	--	--	--
More confident	1.223	0.628	0.419	3.570
I understand Senior Safe goals				
Less confident	--	--	--	--
More confident	0.627	0.403	0.149	2.639
I am committed to achieving Senior Safe goals				
Less confident	--	--	--	--
More confident	10.798	0.004	1.316	88.627
I have clearly defined Senior Safe responsibilities				
Less confident	--	--	--	--
More confident	1.802	0.260	0.469	6.915
I am adequately trained for Senior Safe				
Less confident	--	--	--	--
More confident	0.957	0.936	0.239	3.839
I feel empowered to suggest what does/does not work with Senior Safe				
Less confident	--	--	--	--
More confident	2.360	0.061	0.725	7.682
**Senior Safe Effectiveness** **(AIC = 12.869, BIC = 18.982)**				
Senior Safe helps reduce OTC medication misuse				
Less confident	--	--	--	--
More confident	12.893	**<0.001**	4.744	35.039
**Pharmacy Staff Role** **(AIC = 13.427, BIC = 19.461)**				
Pharmacist	--			
Technician	2.062	0.035	0.852	4.988

**Table 3:** Multivariate Logistic Regression Effects for Multi-Domain Model.

			99% CI	
(AIC = 30.363, BIC = 42.537)	Odds Ratio	Significance Level	Lower	Upper
Senior Safe is well-integrated into leadership operations				
Less confident	--	--	--	--
More confident	5.606	**<0.001**	1.768	17.773
I am committed to achieving Senior Safe goals				
Less confident	--	--	--	--
More confident	6.004	0.031	0.706	51.061
Senior Safe helps reduce OTC medication misuse				
Less confident	--	--	--	--
More confident	8.217	**<0.001**	2.675	25.240

## Data Availability

The raw data supporting the conclusions of this article will be made available by the authors on request.

## Supplementary Materials

The following supporting information can be downloaded at https://www.mdpi.com/article/10.3390/pharmacy13060174/s1, File S1: Senior Safe^TM^ Survey.
