# Supplementary material for: “But Will It Last?”: Examining How Pharmacy Staff Perceptions Influence Beliefs About the Sustainability of a Pharmacy-Based Intervention Targeting Older Adult Over-the-Counter (OTC) Medication Misuse
Source: Pharmacy (Basel). 2025 Dec 1;13(6):174. doi: 10.3390/pharmacy13060174 (PMC12736813; doi:10.3390/pharmacy13060174)
Supplement: Supplementary file 1 [file pharmacy-13-00174-s001.zip › pharmacy-3984083-supplementary.pdf]

Senior Safe™ Survey

We are conducting a survey to help us better understand what you think about Senior Safe™ in Aurora Pharmacies. We invite you to participate in this survey because you are a pharmacist or technician in an Aurora Pharmacy that now uses Senior Safe.

The survey consists of 25 brief items that are primarily multiple choice. Completing the entire survey will take fewer than 5 minutes. Participation in this survey is voluntary. No identifying information will be collected to help ensure anonymity and confidentiality of your responses. Risks may include psychological stress in completing the survey and the potential for breach of confidentiality.

For any question, you can choose “prefer not to answer.” Even if you start the survey, you are not required to complete it. You can stop at any time. The last question in the survey asks if you would like to receive a $10 Amazon electronic gift card. If you would like to receive this incentive, please follow survey instructions to enter an e-mail where a redemption code can be sent to you.

We greatly appreciate your participation in this important work.

Thank you.

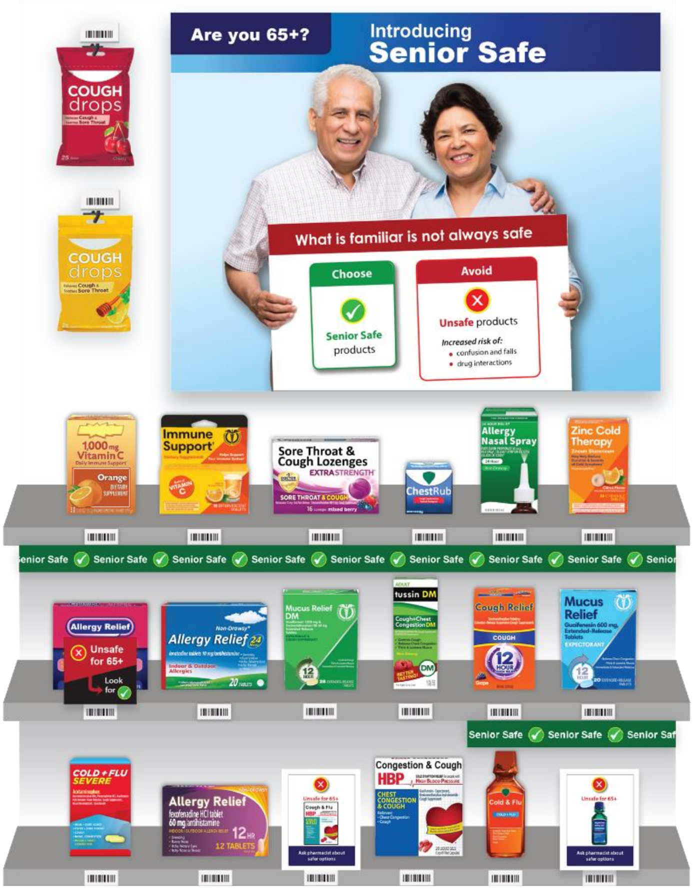

	Not at all	Somewhat	Very much	Unsure	Prefer not to answer
**This first set of questions is meant to evaluate your beliefs about how Aurora Pharmacy Leadership has supported Senior Safe. For the purpose of this survey, Aurora Pharmacy Leadership includes a Manager of Pharmacy Retail Operations (MPO) or the Director of Retail Pharmacy Operations (DPO)**					
1. Senior Safe is part of the mission of **Aurora Pharmacy Leadership**	⊕	⊕	⊕	⊕	⊕
2. Senior Safe is well-integrated into the operations of **Aurora Pharmacy Leadership**	⊕	⊕	⊕	⊕	⊕
3. **Aurora Pharmacy Leadership** supports the goals of Senior Safe	⊕	⊕	⊕	⊕	⊕
4. **Aurora Pharmacy Leadership** clearly communicates the goals of Senior Safe	⊕	⊕	⊕	⊕	⊕
5. **Aurora Pharmacy Leadership** asks pharmacy staff about what works or does not in Senior Safe	⊕	⊕	⊕	⊕	⊕
**This next set of questions is meant to evaluate your perceptions about how <u>pharmacy staff</u> or your <u>Pharmacy Supervisor</u> has supported Senior Safe. For the purpose of this survey, Pharmacy Supervisor is your local site leader at your primary pharmacy/RD site.**					
6. The **Pharmacy Supervisor** supports staff in maintaining Senior Safe	⊕	⊕	⊕	⊕	⊕
7. There are usually enough pharmacy staff to achieve the goals of Senior Safe	⊕	⊕	⊕	⊕	⊕
8. Obstacles were encountered after Senior Safe was put in place	⊕ Yes	⊕ No		⊕	⊕
8a. I or other pharmacy staff removed obstacles to Senior Safe when they arose (ITEM SKIPPED IF NO OBSTACLES WERE ENCOUNTERED)	⊕	⊕	⊕	⊕	⊕
8b. The **Pharmacy Supervisor** removed obstacles to Senior Safe when they arose (ITEM SKIPPED IF NO OBSTACLES WERE ENCOUNTERED)	⊕	⊕	⊕	⊕	⊕
**This next set of questions is meant to evaluate your perceptions about, and your role in, Senior Safe.**					
9. I understand the goals of Senior Safe	⊕	⊕	⊕	⊕	⊕
10. I am committed to achieving the goals of Senior Safe	⊕	⊕	⊕	⊕	⊕
11. I have clearly defined responsibilities related to Senior Safe	⊕	⊕	⊕	⊕	⊕

12. I am adequately trained for Senior Safe ⊕ ⊕ ⊕ ⊕ ⊕

13. I feel empowered to suggest things about what works or does not in Senior Safe ⊕ ⊕ ⊕ ⊕ ⊕

14. I am confident that Senior Safe will still be active in five (5) years ⊕ ⊕ ⊕ ⊕ ⊕

This final set of questions about Senior Safe is meant to evaluate your beliefs about its ability to reduce OTC misuse, as well as other issues that you consider important.

15. In my opinion, Senior Safe helps reduce OTC medication misuse ⊕ ⊕ ⊕ ⊕ ⊕

16. Is there anything else about Senior Safe that is important for us to know? ⊕Yes ⊕ No

17. What else about Senior Safe is important for us to know? [free text]

Demographics

18. Please select your primary site::

□ Traditional pharmacy
□ Remote dispensing site
□ Prefer not to answer

19. Please select the size of your primary pharmacy site (determined by the weekly number of prescriptions filled):

□ 0 to 500 prescriptions filled
□ 501 to 1,200 prescriptions filled
□ 1,201 or more prescriptions filled

□ Unsure

□ Prefer not to answer

20. In some pharmacies, there is not sufficient room to display OTC medications so that they are accessible to patients. Are **<u>ALL</u>** of your OTC pain, cough/cold, and sleep medications located behind the pharmacy counter so that patients must ask pharmacy staff to retrieve them?

□ Yes
□ No
□ Prefer not to answer

21. Please select your current role:

□ Pharmacist
□ Pharmacy Technician
□ Prefer not to answer

20a. How many years have you worked as a pharmacist at Aurora Pharmacies? [free text]

20b. How many years have you worked as a technician at Aurora Pharmacies? [free text]

22. What is your age?

□ _____ years old
□ Prefer not to answer

23. What is your gender?

□ Female
□ Male
□ Prefer to self-describe ____________
□ Prefer not to answer

24. Are you of Hispanic or Latino origin or descent?

□ Yes, Hispanic or Latino
□ No, not Hispanic or Latino

- ☐ Prefer not to answer

25. What is your race? {May choose one or more)
    - ☐ White
    - ☐ Black or African American
    - ☐ Asian
    - ☐ Native Hawaiian or Other Pacific Islander
    - ☐ American Indian or Alaska Native
    - ☐ Other
    - ☐ Prefer not to answer

Survey Payment

26. Please indicate if you would like to receive a $10 Amazon electronic gift card for completing this survey.
    - ☐ Yes
    - ☐ No

To receive a $10 Amazon electronic gift card, please provide your e-mail *(supplying your e-mail is for purpose of receiving the gift card only, and will not be forwarded or used to contact you about this or any topic*).

[free text]

Thank you for completing this survey. Please click the “Submit” button to end the survey.
